# First-interview response patterns of intensive longitudinal psychological and health data

**DOI:** 10.1177/13591053241235751

**Published:** 2024-03-05

**Authors:** Shelley A Blozis

**Affiliations:** University of California, Davis, USA

**Keywords:** intensive longitudinal data, location scale mixed-effects models, patient-reported outcomes, self-report data

## Abstract

Self-report data are essential in health psychology research where an individual’s perception is critical to understanding one’s health and psychological status. Intensive data collection over time, including daily diary assessments, is necessary in understanding within- and between-person variability in health and psychological processes over time. An “initial elevation or latent decline” (IELD) effect, inherent of self-report data, is increasingly acknowledged in the social psychology literature, but awareness of this effect in health psychology research is lacking, particularly in studies that emphasize within- and between-person variability in self-reports. The IELD effect is a pattern in which responses tend to be more extreme at the initial interview relative to subsequent responses. This paper illustrates the impact of IELD in applications of mixed-effects models based on observational self-reports and concludes that researchers take such effects into account in data analysis or in the research designing phase to help mitigate such effects.

Self-reports of individuals’ perceptions of their health and psychological status are inherent to understanding health outcomes (e.g. Basch et al., 2017; Bigatti et al., 2024; Cheng et al., 2021; Fasczewski et al., 2020; Pulcu, 2016). Patient self-reports, for example, are critical for patient assessment and monitoring of health status (Bourgeois et al., 2007). Measured over time, however, self-reports are known, through social psychology research, to show patterns in which responses at the initial assessment are followed by a lessening in severity with repeated measures, independent of a research manipulation or treatment effect, such as mean severity that lessens following the first assessment (Knowles et al., 1996; Robins, 1985; Sharpe and Gilbert, 1998; Windle, 1954). Researchers term this an “attenuation effect” (Jensen et al., 1999; Lucas et al., 1999; Piacentini et al., 1999), but experimental research investigating self-reports of internal states and behaviors provides compelling evidence that the pattern is likely due to an initial response bias and not to true change in the outcome (Shrout et al., 2017). This observed pattern in subjective reports, termed “initial elevation or latent decline” (IELD) effect, has important implications in repeated measures and longitudinal investigations, including those involving panel and daily diary assessments, as well as treatment and observational studies, but accounting for these effects is not routine in applications of statistical models that aim to characterize self-reports in health psychology research.

To understand IELD effects, Shrout et al. (2017) carried out four experiments, three of which involved daily diary self-reports of affect, anxiety and time spent studying for an impending exam deemed stressful to participants and one study of the natural progression of affect and anxiety assessed bimonthly in first-year college students. Within studies, participants were assigned to different entry points into a survey study. In one, for example, participants were randomly assigned to a 14-day assessment where the starting day ranged from 2 to 9 days before a scheduled exam. Across studies, no matter the timing of entry into the survey study, group mean responses were more extreme on the first survey day relative to other days. This pattern of a more extreme mean response for the initial assessment indicated a response bias for the first exposure to the survey as the source of the response pattern, with a stronger bias for more subjective (e.g. affect) relative to more objective outcomes (e.g. time spent studying) and for daily diary relative to panel data. Further, in the daily diary studies involving a common exam and where assessments spanned beyond 1 week, within-group comparisons of the mean response on the first assessment relative to the mean response taken 1 week later (effectively controlling for day of the week) showed greater severity of response at the initial assessment no matter the planned onset of the survey. That is, the initial elevation effect was apparent no matter if the assessments began in advanced of the common exam, near the time of the exam or after.

The field of health psychology notably relies on self-report data, and to understand change or development in health outcomes, repeated-measures study designs are essential. Further, documenting within- and between-person variation in self-report data over time is particularly important to understand individual differences. Beyond the single moment captured from cross-sectional studies, repeated measures data offer insight into a health course or response to treatment over time. Given findings from social psychology research regarding potential for IEDL effects in self-report data, combined with a reliance on repeated measures study designs in health psychology, a critical look at the potential for IEDL effects is needed, especially in applications of statistical models that focus on within- and between-subject response variability.

Mixed-effects models place emphases on the individual and the population and are valued for how they summarize central tendencies in responses across individuals, while also addressing individual differences in data observed over time. Aspects of data that are shared across individuals are referred to as fixed effects, and aspects that vary from person to person are referred to as random effects. For example, Brick et al. (2019) studied the readiness of individuals to avoid a high-fat diet and applied a model where a fixed effect summarized the average rate of change in readiness and a random effect allowed the rate of change to vary between individuals. Many similar applications have appeared in health psychology research (Fortier et al., 2012; Highland et al., 2022; Lenne and Mann, 2020; Pinto-Gouveia et al., 2015). In some applications, for example, a study may include a baseline assessment with subsequent measures following implementation of a treatment to evaluate patient responses to treatment relative to baseline levels (e.g. Serlachius et al., 2016), or repeated measures from an observational study can be used to understand individual differences in responses as they naturally evolve (e.g. Cummings et al., 2017).

In any case, a response bias in the initial assessment, such as a baseline assessment in an experiment or the first assessment in a series of observational assessments, can have unintended consequences for statistical inference. This paper considers the impact of IELD effects on self-report measures obtained from a large daily diary study. Daily diary and other intensive data collections permit comparisons of responses taken at an initial assessment with those that follow soon after, unlike longitudinal studies where assessments are spaced relatively far apart in time, such as annually. The goal is to understand the potential impact of IELD effects in inferences drawn from analyses of variables used in health psychology research. Specifically, IELD effects on measures of central tendency, as well as score variability, are estimated by comparing aspects of a select set of self-report measures on the first day of a daily diary study to the eighth day of the series, thus permitting a test of IELD while controlling for the day of the week. As both central tendencies and score variation are important indicators of health (e.g. Conroy et al., 2016), attention to both aspects of data is necessary. Evidence of IELD effects in both aspects of observational data where no such effects are anticipated would raise concerns about a need to considers such effects in statistical analyses.

The remainder of paper is as follows: Self-report measures motivating the current investigation and its research objectives are presented. Next, statistical models selected for application to these measures are presented and applied to the measures. Findings and implications from the analysis of the data are discussed.

## Motivating data

### Data description

This study involves secondary analysis of publicly available data from the Midlife in the United States (MIDUS) study series; individuals are not identifiable, and the authors’ institution does not require ethics review of studies using such data. From the first wave (MIDUS 1, Brim et al., 1995–1996), participants were selected to be nationally representative of the U.S. population using a random digit-dialing telephone method. The series follows a measurement burst design (Nesselroade, 1991) with eight consecutive daily interviews nested within waves of the larger study. Specifically, for MIDUS 1, a subset of participants was selected at random for a daily diary study, with goals to assess the daily experiences of adults, especially with regard to stressful events. This design repeated for two subsequent waves, namely MIDUS 2 (Ryff and Almeida, 2004–2009) and MIDUS 3 (Ryff and Almeida, 2017–2019). The current study uses diary data from MIDUS 2 and MIDUS 3, with data collection spanning between 2004–09 and 2017–19, respectively. These waves were selected because they shared identical survey items of interest here. Data were collected through a telephone interview, with each interview relating to the previous 24 hours. The day of the week of the first interview varied across individuals. Approximately 30 participants were surveyed each week in each wave; data collection spanned across seasons. Data for participants of the MIDUS “core sample,” “city oversample” and the “core Milwaukee sample” who participated in the daily projects in MIDUS 2 (*n* = 1321 subjects) and MIDUS 3 (*n* = 726 subjects, with a reduction in sample size due to attrition) are used here. Also studied is the independent sample of *n* = 782 participants of MIDUS Refresher (Ryff and Almeida, 2012–2014), with data collection spanning between 2012-14. Variables identical to those selected from MIDUS 2 and 3 were used to replicate those analyses.

Similar to Shrout et al. (2017), selected variables reflect a variety of attributes: a continuous measure of positive affect, a binary symptom report of pain (yes or no), a continuous measure of time spent on leisure and two measures relating to symptoms of fatigue. Positive affect was measured by averaging responses to 13 questions using a 5-point scale (0 = none of the time; 1 = a little of the time; 2 = some of the time; 3 = most of the time; 4 = all of the time). Higher scores reflect greater affect levels. Participants were asked to report time spent (in minutes) on leisure (converted to hours for analysis). For pain, participants were asked if they experienced a joint pain (yes or no). For fatigue, participants were asked if fatigue was experience (yes or no); if a positive report was given, the participant was asked to rate the severity on a scale from 1 (very mild) to 10 (very severe). Indeed, accounting for the severity of symptom, in addition to its daily frequency, may improve characterization of daily symptoms (Schneider and Stone, 2014)) Using both responses, a semi-continuous measure of fatigue severity was created (described later). Time spent on leisure and fatigue severity are analyzed as semi-continuous to distinguish between whether or not a positive report was made and the duration (time spent on leisure) or severity (fatigue) conditional that positive reports were made. Continuous reports of time spent on leisure were positively skewed and log-transformed (base 10) prior to analysis. Variables were analyzed individually using all available data. Missing data are assumed to be missing at random.

### Research objectives

Aspects of the MIDUS study are pertinent to the current study. First, the study is based on an observational design, and as such, there is no expectation that daily means would show any particular pattern according to the order of interview days, and in particular, a mean difference between the first interview and those that followed. Second, data were collected across 8 consecutive days, permitting comparisons between reports on the first day and 1 week later to assess IELD effects while controlling for day of the week. Third, given the measurement burst design of MIDUS 2 and 3, the data permit examination of whether IELD effects are present at both longitudinal waves. Evidence of IELD effects at the second wave would suggest persistence of these effects several years later. Finally, the availability of data from the MIDUS Refresher allows study of whether results from analyses of MIDUS 2 and 3 data replicate to an independent sample. With these, the research objectives are to test for IELD effects on the parameters of statistical models applied to the daily measures.

## Mixed-effects models for selected daily measures

Mixed-effects models are described for the analysis of the selected variables, beginning with a linear mixed-effects model for the continuous measure of positive affect, followed by a logistic mixed-effects model for the binary indicator of pain, and ending with two-part mixed-effects models for time spent on leisure and fatigue. To study the effects of IELD on model parameters, indicator variables were created to denote interview days 2–8, with day 1 serving as the reference day. IELD effects were estimated as differences in particular aspects of the responses between day 1 and day 8. Effects of IELD were considered for parameters characterizing the mean response and measures of within- and between-subject variability. The models applied to each variable are described next, followed by a summary of results.

### A linear mixed-effects model for positive affect scores

For positive affect, let *y_ti_* be the score for subject *i* at day *t*, where *i* = 1,. . ., *N*, *N* is the number of subjects, *t* = 1,. . ., *n*_i_, and *n*_i_ is the number of measures for *i*. The model for *y_ti_* was

(1) $$y_{ti}=\beta_{0}+\beta_{1}day_{2}+\ldots +\beta_{7}day_{8}+b_{0i}+e_{ti},$$

where $\beta_{0}$ is the expected response at day 1, $\beta_{1},\ldots ,\beta_{7}$ are effects of days 2–8, $b_{0i}$ is a random subject effect assumed to be normally distributed with mean 0 and variance $\phi_{b}^{2}$ , and $e_{ti}$ is the residual. One of three effects of IELD was estimated by $\beta_{0}-\beta_{7}$ to test for a mean difference in positive affect between days 1 and 8. Letting $e_{i}=\left(e_{1i},\ldots ,e_{n_{i}}\right)\prime$ contain the residuals from (1), $e_{i}$ is assumed to be normally distributed with mean **0** and covariance matrix $\Theta_{e}$ ; the residuals were assumed to be normally distributed and independent between days.

The variance of the random subject effect $\phi_{\;\;b}^{2}$ quantifies the extent of individual differences in the expected value of $y_{i}$ adjusting for the effect of day 1. For positive affect, this variance describes between-subject differences in daily mean affect across the study period. It is common to assume that the variance of a random subject effect is constant across occasions and subjects where the goal is simply to estimate this source of variation in the outcome, but a variance may also be a function of covariates. Sandhu and Leckie (2016), for example, used a mixed-effects model to study pain experiences over time for girls and boys and reported that the variance of the random subject effect (that represented individual-level means) was greater for girls relative to boys, indicating greater differences in pain levels among girls relative to differences among boys. Here, the variance of $b_{0i}$ was modeled by (cf: Hedeker and Nordgren, 2012)

$$\phi_{b}^{2}=\exp\left(\kappa_{b0}+\kappa_{b1}day_{2}+\ldots +\kappa_{b7}day_{8}\right),$$

where $\kappa_{b0}$ exponentiated is the random subject effect variance at day 1, and $\kappa_{b1},\ldots ,\kappa_{b7}$ are effects of interview days 2–8. The second of the three effects of IELD was measured as the difference of $\kappa_{b0}-\kappa_{b7}$ to test for a difference in the variance between days 1 and 8.

The set of residuals $e_{i}$ reflect differences between the fitted values according to a model and the observed scores. Although residuals are often assumed to be independent between occasions with constant variance across time and subjects, these assumptions may be relaxed. The assumption of independence can be relaxed, such as by permitting the residuals to correlate between occasions (see Singer and Willett, 2003). The assumption of homogeneity of variance can be relaxed by allowing the residual variance to be a function of covariates and by including a random scale effect (cf: Hedeker et al., 2008; Blozis, 2022 in what is called a mixed-effects location scale (MELS) model. For the positive affect model, the residuals were assumed to be independent between days, but the residual variance was assumed to differ by the interview day, making it was possible to test for an IEDL effect on this variance:

$$\sigma_{\varepsilon_{ti}}^{2}=\exp\left(\tau_{0}+\tau_{1}day_{2}+\ldots +\tau_{7}day_{8}+c_{i}\right),$$

where the exponentiated value of $\tau_{0}$ is the residual variance at day 1 for an individual whose random scale effect $c_{i}$ is 0, and $\tau_{1},\ldots ,\tau_{7}$ are effects of interview days 2–8. A positive effect would indicate greater within-subject variation on a given day and a negative effect would indicate less variation. The random scale effect $c_{i}$ , assumed to be independent and normally distributed between subjects with mean 0 and variance $\phi_{c}^{2}$ , permits the residual variance to differ between subjects after adjusting for day effects. Thus, $c_{i}$ permits the within-subject residual variance (i.e. within-subject score variation about an individual’s mean across days) to differ between individuals. Given the random subject effect $b_{0i}$ and scale effect $c_{i}$ , the variance-covariance matrix of these random effects is

$$\Phi =\left[\begin{array}{cc} \phi_{b}^{2} & \phi_{bc}\\ \phi_{cb} & \phi_{c}^{2} \end{array}\right],$$

where $\phi_{cb}$ is the covariance between the two random effects.

### A logistic mixed-effects model for pain reports

Raudenbush and Bryk (2002) provide about a logistic mixed-effects model and its interpretation for repeated binary responses. A model for the binary measures of pain is described here. Let $y_{ti}=1$ if pain was reported on day *t* and $y_{ti}=0$ if not. Given repeated measures, let $y_{i}=\left(y_{1i},\ldots ,y_{n_{i}}\right)$ . Let $\eta_{ti}$ be the logit of the probability that the individual reported pain (i.e. $P\left(y_{ti}=1\right)$ ):

$$\eta_{ti}=\log\left[P\left(y_{ti}=1\right)/\left(1-P\left(y_{ti}=1\right)\right)\right],$$

and assume that $\eta_{ti}$ follows a mixed-effects model:

$$\eta_{ti}=\alpha_{0}+\alpha_{1}day_{2}+\ldots +\alpha_{7}day_{8}+a_{i},$$

where $\alpha_{0}$ is the expected logit at day 1, $\alpha_{1},\ldots ,\alpha_{7}$ are effects of interview days 2–8, and $a_{i}$ is a random subject (assumed to be normally distributed with a mean of 0 and variance $\phi_{a}^{2}$ ) that permits the conditional logit to vary between individuals. The magnitude of $\phi_{a}^{2}$ corresponds to the degree of individual differences in the conditional logit. One of two IELD effects for pain measures was estimated by $\alpha_{0}-\alpha_{7}$ to test for a difference in logits between days 1 and 8. Similar to the random subject effect variance of the positive affect model, the variance of $a_{i}$ was modeled as

$$\phi_{a}^{2}=\exp\left(\kappa_{a0}+\kappa_{a1}day_{2}+\ldots +\kappa_{a7}day_{8}\right),$$

where $\kappa_{a0}$ exponentiated is the variance at day 1, and $\kappa_{a1},\ldots ,\kappa_{a7}$ are effects of interview days 2–8. The second IELD effect was evaluated by $\kappa_{a0}-\kappa_{\alpha 7}$ to test the difference in variances between days 1 and 8. In other words, this effect permitted a test of whether between-subject variation in the person-specific logits differed between day 1 and day 8.

### A two-part mixed-effects model for leisure time and fatigue severity

Semi-continuous outcomes represent to a special type of variable with features characteristic of some variables studied in health psychology and behavioral medicine research, particularly variables that include 0 to denote the absence of a measured behavior or symptom (e.g. Baldwin et al., 2016; Conroy et al., 2016; Gilchrist et al., 2020; Ruf et al., 2023; Wen et al., 2018). A semi-continuous response is defined by a combination of a discrete response (usually 0) and continuous values (e.g. behavior or symptom intensity when present). A two-part mixed-effects model was developed for repeated measures of semi-continuous outcomes (Olsen and Schafer, 2001; Tooze et al., 2002). Conroy et al. (2016), for example, apply a two-part multilevel model to daily measures of physical activity to simultaneously model a daily indicator of an individual’s activity engagement (yes or no) and the duration of activity conditional on any positive amount of time spent. The model has two parts: The first is for the binary response that indicates the presence or absence of the outcome, such as whether or not an individual experienced fatigue; the second is for the positive and continuous response, such as fatigue severity, conditional that a positive report was made.

In fitting a two-part model to time spent on leisure, let $y_{ti}$ be the reported time spent on day *t* for subject *i*. From $y_{ti}$ , two variables are defined: $r_{ti}=1$ if any time was spent and $r_{ti}=0$ if no time was spent (if $y_{ti}$ is missing, then $r_{ti}$ is missing), and $s_{ti}=y_{ti}$ if any time was spent ( $s_{ti}$ is missing otherwise). Given repeated, let $r_{i}=(r_{1i},\ldots ,r_{n_{i}})$ and $s_{i}=(s_{1i},\ldots ,s_{m_{i}})$ where $n_{i}$ is the number of responses in $y_{i}$ and $m_{i}$ is the number of responses in $y_{i}$ greater than 0. For the binary response $r_{ti}$ , let $\eta_{ti}$ be the logit of the probability that the individual reported any time spent (i.e. $P\left(r_{ti}=1\right):$

$$\eta_{ti}=\log\left[P\left(r_{ti}=1\right)/\left(1-P\left(r_{ti}=1\right)\right)\right],$$

and assume that $\eta_{ti}$ follows a mixed-effects model:

$$\eta_{ti}=\alpha_{0}+\alpha_{1}day_{2}+\ldots +\alpha_{7}day_{8}+a_{i},$$

where interpretation of the model follows that given for the logistic mixed-effects model used to describe binary indicators of pain.

For the second model part, a linear mixed-effects model for positive reports of time spent, $s_{ti}$ , is

$$s_{ti}=\beta_{0}+\beta_{1}day_{2}+\ldots +\beta_{7}day_{8}+b_{0i}+e_{ti},$$

where interpretation of the model follows that given earlier for the linear mixed-effects model applied to positive affect scores. The two model parts are then joined through a covariance between the random subject effects of each model part:

$$\Phi =\left[\begin{array}{cc} \phi_{a}^{2} & \\ \phi_{ba} & \phi_{b}^{2} \end{array}\right],$$

where $\phi_{ba}$ is the covariance between the individual-level logit from the binary response model and the conditional mean time spent from the continuous response model. A positive covariance, for example, would indicate that a higher log odds of reporting time spent corresponds to a higher mean time spent on days when any positive amount of time was spent. For measures of fatigue, a similar two-part mixed-effects model was applied. Specifically, the first question that asked if fatigue was experienced (yes or no) served as the binary response for the first model part, and the subsequent question that asked about the severity of fatigue conditional that a positive report was made served as the continuous response to the second model part.

### Model estimation

A linear mixed-effects model may be estimated using a statistical software program intended for mixed-effects models, such as the R package *lme4* (Bates et al., 2015). If a model includes a nonlinear function, such as those here that use exponential functions to model variances, software for fitting nonlinear mixed-effects models, such as SAS PROC NLMIXED (Hedeker et al., 2008), MIXREGLS (Hedeker and Nordgren, 2013) or SAS IML (Blozis, 2022). SAS (version 9.4) PROC NLMIXED was used to carry out maximum likelihood estimation of the models. Scripts for data analysis are in Supplemental Materials. For each outcome, model fitting started with a simple model that excluded covariates, building up to the particular model selected and described earlier (Kiernan et al., 2012). Starting values for parameter estimates in the simplest model were based on descriptive statistics of the data. Estimates from simpler models were used for starting values as models increased in complexity.

## Results

### Effects of IELD

Estimated IELD effects on model parameters describing expected values and variation in responses as measured by differences between day 1 and day 8 are in Table 1. For positive affect, IELD estimates pertaining to the mean response from MIDUS 2 (estimate = 0.04, CI: 0.01 0.06), MIDUS 3 (estimate = 0.07, CI: 0.04 0.11) and MIDUS Refresher (estimate = 0.09, CI: 0.06 0.13) indicate slightly elevated mean levels on the first interview day relative to 1 week later. Estimates pertaining to the variance of the random subject effect indicate less between-subject variability in subject-specific means on day 1 versus 1 week later for MIDUS 2 (estimate = −0.26, CI: −0.36, −0.15), MIDUS 3 (estimate = −0.23, CI: −0.33, −0.14) and MIDUS Refresher (estimate = −0.24, CI: −0.32, −0.15). These estimates indicate greater similarity in the subject-specific means on the first interview day relative to 1 week later. Conversely, estimates of the within-subject residual variance (when the random scale effect is equal to 0) indicate greater within-subject variation on the first interview day relative to 1 week later for MIDUS 2 (estimate = 0.50, CI: 0.30, 0.70), MIDUS 3 (estimate = 0.51, CI: 0.31, 0.71) and MIDUS Refresher (estimate = 0.61, CI: 0.42, 0.79). In other words, there was greater within-individual variability in responses on the first interview day relative to 1 week later.

**Table 1.** ML estimated effects of IELD (Day 1 vs Day 8) on fixed-effects and variance parameters.

Parameter	Level	Logit	$\phi_{b}^{2}$	$\phi_{a}^{2}$	$\sigma_{\varepsilon }^{2}$
MIDUS 2	Est (95% CI)	Est (95% CI)	Est (95% CI)	Est (95% CI)	Est (95% CI)
Positive affect	0.04 (0.01, 0.06)		−0.26 (−0.36, −0.15)		0.50 (0.30, 0.70)
Pain (yes/no)		2.1 (1.6, 2.6)		−0.95 (−1.4, −0.53)	
Leisure^a^	0.22 (0.17, 0.27)	0.12 (−0.41, 0.66)	−0.46 (−0.69, −0.23)	−0.80 (−1.6, −0.03)	0.23 (0.09, 0.37)
Fatigue	0.83 (0.56, 1.1)	1.6 (1.3, 2.0)	−0.35 (−0.78, 0.07)	−0.80 (−1.3, −0.33)	0.38 (0.05, 0.70)
MIDUS 3					
Positive affect	0.07 (0.04, 0.11)		−0.23 (−0.33, −0.14)		0.51 (0.31, 0.71)
Pain (yes/no)		2.0 (1.4, 2.6)		−0.97 (−1.6, −0.38)	
Leisure^a^	0.31 (0.25, 0.38)	0.41 (−0.73, 1.6)	−0.37 (−0.59, 0.14)	−0.17 (−1.2, 0.82)	0.31 (0.11, 0.50)
Fatigue	0.43 (0.11, 0.75)	2.0 (1.3, 2.6)	0.01 (−0.44, 0.45)	−1.1 (−1.7, −0.42)	0.13 (−0.34, 0.59)
MIDUS Refresher					
Positive affect	0.09 (0.06, 0.13)		−0.24 (−0.32, −0.15)		0.61 (0.42, 0.79)
Pain (yes/no)		2.0 (1.2, 2.9)		−0.59 (−1.2, 0.001)	
Leisure^a^	0.23 (0.16, 0.30)	−0.17 (−1.2, 0.91)	−0.12 (−0.32, 0.09)	−1.4 (−2.5, −0.32)	0.10 (−0.09, 0.29)
Fatigue	0.55 (0.27, 0.82)	1.9 (1.3, 2.4)	−0.36 (−0.71, 0.00)	−0.92 (−1.5, −0.30)	0.41 (−0.04, 0.86)

IELD effects reflect differences between the effects of Day 1 and Day 8 (estimate = Day 1 effect - Day 8 effect). A linear mixed-effects models was fit to positive affect scores. A logistic mixed-effects model was fit to binary measures of pain symptoms. Two-part mixed-effects models were fit to time spent on leisure and fatigue. Est = estimate.

a Continuous scores were log-transformed (base 10). $\phi_{b}^{2}$ and $\phi_{a}^{2}$ are the variances of random subject effects relating to binary and continuous models, respectively. $\sigma_{\varepsilon }^{2}$ is the within-subject variance when the random scale effect $v_{i}$ = 0.

IELD effects on the expected logit for pain from MIDUS 2 (estimate = 2.1, 95% CI: 1.6, 2.6), MIDUS 3 (estimate = 2.0, 95% CI: 1.4, 2.6), and MIDUS Refresher (estimate = 2.0, 95% CI: 1.2, 2.9) indicate a greater likeliness to report pain on the first interview day relative to 1 week later. IELD effects on the variance of the random subject, $\phi_{a}^{2}$ , indicate (except for scores from MIDUS Refresher) less between-subject variability on day 1 versus 1 week later. Specifically, the estimates from MIDUS 2 (estimate = −0.95, 95% CI: −1.4, −0.53) and MIDUS 3 (estimate = −0.97, 95% CI: −1.6, −0.38) indicate greater similarity in the subject-specific logits on the first interview day relative to 1 week later.

IELD effects on the expected logit for time spent on leisure for MIDUS 2 (estimate = 0.12, 95% CI: −0.41, 0.66), MIDUS 3 (estimate = 0.41, 95% CI: −0.73, 1.6), and MIDUS Refresher (estimate = −0.17, 95% CI: −1.2, 0.91) indicate no clear direction of the effects. IELD effects on the variance of the random subject effect, $\phi_{a}^{2}$ , however, indicate less between-subject variability in logits on day 1 versus 1 week later for MIDUS 2 (estimate = −0.95, 95% CI: −1.4, −0.53), MIDUS 3 (estimate = −0.97, 95% CI: −1.6, −0.38), and MIDUS Refresher (estimate = −1.4, 95% CI: −2.5, −0.32). For the positive measures of time spent, IELD effects indicate elevated mean responses on the first day versus 1 week later for MIDUS 2 (estimate = 0.22, CI: 0.17, 0.27), MIDUS 3 (estimate = 0.31, CI: 0.25 0.38) and MIDUS Refresher (estimate = 0.23, CI: 0.16, 0.30). IELD effects on $\phi_{b}^{2}$ indicate less between-subject variability in subject-specific means on day 1 relative to 1 week later for MIDUS 2 (estimate = −0.46, 95% CI: −0.69, −0.23), but the direction of the effect is unclear for MIDUS 3 (estimate = −0.37, 95% CI: −0.59, 0.14) and MIDUS Refresher (estimate = −0.12, 95% CI: −0.32, 0.09). Regarding within-subject variability, IELD effects (when the random scale effect is equal to 0) indicate greater within-subject variation on the first interview day relative to 1 week later for MIDUS 2 (estimate = 0.23, CI: 0.09, 0.37) and MIDUS 3 (estimate = 0.31, CI: 0.11, 0.50), but the direction of the effect is unclear for MIDUS Refresher (estimate = 0.10, CI: −0.09, 0.29).

Estimates of the expected logit for fatigue (yes or no) from MIDUS 2 (estimate = 1.6, 95% CI: 1.3, 1.9), MIDUS 3 (estimate = 2.0, 95% CI: 1.3, 2.6), and MIDUS Refresher (estimate = 1.9, 95% CI: 1.4, 2.4) indicate a greater likeliness to report fatigue on the first interview day relative to 1 week later. IELD effects on the variance of the random subject effect, $\phi_{a}^{2}$ , indicate less between-subject variation on day 1 relative to day 8 for MIDUS 2 (estimate = −0.80, 95% CI: −1.3, −0.33), MIDUS 3 (estimate = −1.1, 95% CI: −1.7, 0.42), and MIDUS Refresher (estimate = −0.92, 95% CI: −1.5, −0.30). IELD effects indicate elevated mean responses on day 1 relative to day 8 for MIDUS 2 (estimate = 1.6, CI: 1.3, 2.0), MIDUS 3 (estimate = 2.0, CI: 1.3, 2.6) and MIDUS Refresher (estimate = 1.9, CI: 1.3, 2.4). IELD effects on $\phi_{b}^{2}$ indicate no clear direction in effects on the variation in subject-specific means on day 1 relative to day 8 for MIDUS 2 (estimate = −0.35, 95% CI: −0.78, 0.07), MIDUS 3 (estimate = 0.01, 95% CI: −0.44, 0.45) and MIDUS Refresher (estimate = −0.36, 95% CI: −0.71, 0.00). Conversely, IELD on the within-subject residual variance indicate less within-subject variation on day 1 relative to day 8 for MIDUS 2 (estimate = 0.38, CI: 0.05, 0.70), but the direction of the effect was unclear for MIDUS 3 (estimate = 0.13, CI: −0.34, 0.59) and MIDUS Refresher (estimate = 0.41, CI: −0.04, 0.86).

## Discussion

Self-report data play a critical role in health assessment and monitoring, but they can exhibit an unexpected response pattern relating to the initial assessment. This pattern, known as the “attenuation effect” or the “initial elevation or latent decline” (IELD) effect, has been attributed to an initial response bias rather than true change in outcomes (Anvari et al., 2023; Shrout et al., 2017). Previous experimental research conducted to understand this effect focused on self-reports that included mood and objective behaviors (reported time spent studying for an upcoming exam). Those experiments showed evidence of an initial assessment bias that was most pronounced for more subjective outcomes and for daily diary versus longitudinal studies. Similar patterns are reported here using multiple large samples from an observational study design.

Mixed-effects models developed to analyze repeated measures and longitudinal data address individual differences in response variation by allowing the coefficients of a model, such as an intercept or slope, to vary between individuals. Extensions of these models can be used to address different sources of heterogeneity of variance, including sources that impact the variances of random coefficients or the within-subject residual variance. This article discusses situations in which these models are applied to repeated measures of self-report data where study aims include documentation of mean responses and variability in the outcomes. Using observational data for a variety of outcomes, this study reports evidence of the effects of the first day of a daily interview series where such patterns were not expected given the nature of the study design. Specifically, IELD effects, defined as differences between the first interview day and 8 days later, were evident for measures of central tendency, including the means of continuous variables and the logits of binary variables, as well as measures of variability, including the variances of random effects (i.e. random intercepts that reflected the individual-specific means of continuous variables or logits of binary variables) and the within-subject residual variance (i.e. that variance reflecting conditional score variation about an individuals fitted response across an 8-day period). Results from these analyses naturally depend on the models selected for examining IEDL effects, and alternative statistical models might be considered in practice, such as a latent variable mixed-effects model to address measurement error in self-reports (Nestler, 2020; Blozis, 2022) or the Likert-type scales typically used in self-report measures.

This report illustrates the impact of the first interview day on estimated means and variances of daily diary data and recommends that researchers consider this issue when designing studies that rely on self-reports. For time intensive data collection, including daily diary designs, strategies that include those discussed here and Shrout et al. (2017) might be considered, such as planning for data collection to span at least 8 days to allow for assessment of differences between the first day and 1 week later to estimate IELD effects. In addition to including an indicator of the first assessment in a statistical model or accounting for the interview day (Mosteo et al., 2023), other options include excluding the initial assessment prior to proceeding with data analysis (Heininga et al., 2023). One idea for experimental designs in particular might be to administer two back-to-back baseline assessments to evaluate initial responses that might then be considered for exclusion from a data analysis.

### Implications for health psychology research

Analyzes of self-reports that ranged in their level of subjectivity showed tendencies of mean differences and differences in summary measures of score variability between the first interview day and 1 week later using observational daily diary data where no such differences were anticipated. Initial response biases were evident for mean responses, variability in scores both within- and between-individuals, and the likeliness to report particular outcomes (i.e. pain and fatigue). Given the importance of including individuals’ perceptions of their clinical and health status as important contributors to understanding patient outcomes and treatment experiences (e.g. Opara et al., 2010), analysts might consider the possibility of initial response biases in all aspects of a statistical model, including biases that could impact the mean and variability in responses. Although the data considered here were observational, the results, coupled with those based on experiments reported in (Shrout et al., 2017), pose a source of concern for experimental studies as well, including those involving control-group comparisons, where similar response patterns have the potential to impact estimated clinical effects.

## Supplemental Material

sj-docx-1-hpq-10.1177_13591053241235751 – Supplemental material for First-interview response patterns of intensive longitudinal psychological and health data Supplemental material, sj-docx-1-hpq-10.1177_13591053241235751 for First-interview response patterns of intensive longitudinal psychological and health data by Shelley A Blozis in Journal of Health Psychology
