# Supplementary material for: First-interview response patterns of intensive longitudinal psychological and health data
Source: J Health Psychol. 2024 Mar 5;30(1):65–76. doi: 10.1177/13591053241235751 (PMC11686926; doi:10.1177/13591053241235751)
Supplement: sj-docx-1-hpq-10.1177_13591053241235751 – Supplemental material for First-interview response patterns of intensive longitudinal psychological and health data [file sj-docx-1-hpq-10.1177_13591053241235751.docx]

Supplemental Material

SAS script for using PROC NLMIXED is provided here. Scripts for fitting (1) a mixed-effects model, (2) a logistic mixed-effects model and (3) a two-part mixed-effects model follow.

1. For a normally distributed variable:

proc nlmixed;

m = B2DPOSAV; *specify the response variable name;

pi = arcos(-1); *a value needed to calculate the likelihood function value;

*User provides initial parameter estimates;

parms

b0 2.7908

tau0 -2.2055

alpb -0.4689

sdv 0.9802

Xrhovb -0.3595;

rhovb = tanh(Xrhovb); *a transformation of the correlation coefficient so that estimation is not restricted to the bounds of -1 to 1;

sdb = sqrt(exp(alpb)); *standard deviation of the random intercept;

s2e = exp(tau0+vi*sdv); *within-subject residual variance model;

mu= b0 + bi*sdb; *predicted value of the response;

LL2 = -.5*(((m-mu)**2)/s2e + log(s2e) + log(2*pi)); *likelihood for a normally distributed variable;

Loglik=LL2; *log of the likelihood;

random bi vi ~ normal([0,0],[1,rhovb,1]) subject=m2id;

model m ~ general(Loglik);

1. For a binary response variable:

proc nlmixed;

u=pain_u; *specify the binary response variable name;

*User provides initial parameter estimates;

parms

a0 -4.167

alpa0 3.1019

ax 2.4317

alpax -0.9317;

sda = sqrt(exp(alpa0)); *standard deviation of the random intercept;

ueta = a0 + ai*sda; *predicted value of the response;

expeta = exp(ueta);

p=expeta/(1+expeta);

LL = log((1-p)**(1-u)) + log(p**(u)); *log-likelihood for a binary variable;

random ai ~ normal([0],[1]) subject=MRID;

model u ~ general(LL);

run;

1. a two-part mixed-effects model

proc nlmixed;

u=leisure_u; *specify the binary response variable name;

m=leisure_m; *specify the positive, continuous response variable name;

pi = arcos(-1); *a value needed to calculate the likelihood function value;

*User provides initial parameter estimates;

parms

a0 3.3515

alpa0 1.1636

xrhoba 0.4949

xrhova -0.575

b0 0.829

tau0 -1.0208

alpb0 -1.2187

sdv 0.6344

xrhovb -0.6536;

* transformations of the correlation coefficients so that estimation is not restricted to the bounds of -1 to 1;

rhoba = tanh(Xrhoba);

rhova = tanh(Xrhova);

rhovb = tanh(Xrhovb);

s2e = exp(tau0 + vi*sdv); *within-subject residual variance model;

sda = sqrt(exp(alpa0)); *standard deviation of the random intercept of the logistic model part;

sdb = sqrt(exp(alpb0)); *standard deviation of the random intercept of the linear model part;

*LL1: Binary part;

ueta = a0 + ai*sda;

expeta = exp(ueta);

p=expeta/(1+expeta);

LL1 = log((1-p)**(1-u)) + log(p**(u));

*LL2: Continuous part;

if leisure_u = 1 then do;

mu= b0 + bi*sdb;

LL2 = -.5*(((m-mu)**2)/s2e + log(s2e) + log(2*pi));

end;

if leisure_u = 0 then Loglik=LL1;

else if leisure_u = 1 then Loglik=LL1+LL2;

random ai bi vi ~ normal([0,0,0],[1,

rhoba,1,

rhova,rhovb,1]) subject=MRID;

model leisure_hours ~ general(Loglik);

run;
